# Exploring factors influencing health empowerment in Saudi Arabia: bridging the gap for equitable health utilization

**DOI:** 10.3389/fpubh.2026.1860728

**Published:** 2026-07-10

**Authors:** Maaidah M. Algamdi

**Affiliations:** Community and Psychiatric Health Nursing Department, Faculty of Nursing, University of Tabuk, Tabuk, Saudi Arabia

**Keywords:** gender disparities, health empowerment, health equity, health services utilization, HES-A, public health, Saudi Arabia, sociodemographic factors

## Abstract

**Background:**

Addressing disparities in equitable health service utilization in Saudi Arabia is essential to designing targeted public health interventions. This study aimed to assess levels of health empowerment across sociodemographic variables and to evaluate participation in Vision 2030 health initiatives.

**Methods:**

A cross-sectional quantitative study administered the Arabic version of the Health Empowerment Scale (HES-A) to 420 participants recruited via online social media platforms. Chi-square tests, one-way ANOVA, and multiple linear regression were employed to analyze group-level differences and predictors of health empowerment.

**Results:**

The sample was predominantly male (52.4%), urban-dwelling (69.8%), married (65.0%), and employed (67.6%). Gender was the only significant predictor of HES-A scores in the regression model, accounting for 8.4% of the variance (*R*^2^ = 0.084; β = 0.252, *p* < 0.001). Males reported higher empowerment scores than females (mean 4.041 vs. 3.612). Rural residents reported marginally higher empowerment than urban residents (mean 3.965 vs. 3.782; *F*(1,418) = 4.081, *p* = 0.044). The majority of participants (56.4%) preferred government health facilities, 71.7% maintained an active medical record, and 75.0% used digital health platforms. Cultural norms positively influenced healthcare-seeking behavior in 58.3% of participants.

**Conclusion:**

Gender and residential location are key factors shaping health empowerment in Saudi Arabia. The findings highlight the importance of culturally sensitive, gender-responsive interventions and the expansion of digital health to advance equitable health utilization aligned with Vision 2030 objectives.

## 1 Introduction

Health empowerment is the process of enabling patients to take responsibility for their health by giving them greater authority over matters they consider significant. Prioritizing patient perspectives can ultimately foster empowerment and promote progressive improvements in wellbeing through culturally informed decisions (1–4). Enhancing patient empowerment in healthcare settings involves encouraging individuals to actively participate in decisions that affect their health, encompassing social, cultural, and psychological dimensions (5). According to the World Health Organization (WHO), empowerment is essential for expanding global access to healthcare, reducing performance disparities, and improving availability at the international level (6). Evidence indicates that health empowerment can enhance management of chronic illnesses, improve patient adherence, and increase cost-efficiency (1, 2, 7), positioning it as a central element in patient-centered, equitable therapeutic care (8).

Health empowerment represents a top global priority with significant regional implications. Saudi Arabia has aligned its policies with the Sustainable Development Goals, particularly SDG 3, as an integral component of Vision 2030 (9), a comprehensive national transformation strategy initiated in 2016 to diversify the economy and enhance public services, including healthcare (10). Its health-related objectives include increasing life expectancy from 74 to 80 years, extending preventive care coverage to 80% of the population, privatizing 60% of health services, implementing national digitization of health records, and elevating Saudi women's participation in the healthcare workforce from 25% to 40% (11). According to the Health Sector Transformation Report, considerable progress has been achieved toward these objectives, with digital health platforms now serving tens of millions of beneficiaries and over 97% of populated areas having access to fundamental health services (12).

Within this national context, health empowerment is also shaped by individual sociodemographic characteristics, which collectively constitute the primary focus of the present study. Gender influences empowerment through cultural and systemic factors that may restrict women's autonomy over their health and their access to female healthcare providers (5). Age may influence empowerment through accumulated health experience, counterbalanced by declining digital literacy among older adults (13). The area of residence is significant because rural populations often encounter more substantial geographic and infrastructural obstacles to access specialist and emergency care. Despite recent expansions in the health system, disparities between rural and urban areas continue to persist (14, 15). Income affects access to private healthcare and health information, thereby potentially alleviating financial stress that may impair health-related decisions (16). Furthermore, educational attainment correlates with improved health and e-health literacy, thereby facilitating problem-solving and the critical evaluation of health information (17). Marital status is associated with health empowerment through social support and physiological mechanisms, with potential differences between men and women (18, 19). Employment status offers income, social connections, and frequently health insurance, with documented associations to health-related outcomes (20). Finally, smoking status, chronic illness, and medication use reflect underlying health burdens: Saudi Arabia encounters significant and increasing challenges from chronic diseases such as diabetes, obesity, and hypertension (21, 22), conditions that require continuous informed decision-making and are therefore closely associated with health empowerment. The current study assesses these variables collectively to identify which, if any, serve as independent predictors of health empowerment within the Saudi context.

One concrete policy response is the broader adoption of community-based healthcare professional programs, wherein certified health workers from neighboring communities provide direct care in remote regions. By offering gender-matched, culturally familiar, and continuous rather than episodic care close to patients' residences, such initiatives can mitigate provider shortages, alleviate travel burdens, and foster trust, all while enhancing health literacy through accessible educational formats (23). These measures align with Vision 2030′s overarching commitment to diminishing gender-based social disparities and augmenting women's access to healthcare and education (24–27).

Despite these advancements, substantial disparities continue to exist. Traditional gender roles may influence women's healthcare-seeking behaviors (5). Rural regions have significantly fewer physicians per capita than urban areas, and within these regions, the proportion of female providers is proportionally even lower, thereby intensifying access barriers for women in rural settings (28). At a systemic level, reduced participation of women in the workforce results in lower private insurance coverage among women, consequently increasing dependence on public healthcare facilities (27). Although these gender- and location-based disparities have garnered considerable scholarly attention, the manner in which they interact with other sociodemographic factors—and align with Vision 2030′s broader health-empowerment initiatives—remains inadequately understood.

The present study addresses this gap by measuring health empowerment across the full spectrum of previously delineated sociodemographic dimensions, in conjunction with engagement with Vision 2030 health initiatives. Specifically, this study aims to: (i) evaluate health empowerment among diverse sociodemographic groups; (ii) identify principal predictive factors; and (iii) assess participation in Vision 2030 health programs. In so doing, the study provides valuable insights to inform gender-equity policies and to mitigate rural–urban health disparities, thereby supporting healthcare delivery in alignment with the nation's transformation objectives.

## 2 Materials and methods

### 2.1 Study design

A cross-sectional quantitative study design was employed to examine differences in health empowerment scores across sociodemographic groups (including age, gender, income, residence, marital status, education, employment, smoking, chronic illness, and medication use) using the Arabic version of the Health Empowerment Scale (HES-A).

### 2.2 Setting and recruitment

The questionnaire was distributed through online social media platforms—including Platform X, WhatsApp, and Snapchat—to ensure accessibility for individuals of all genders across all geographic regions of Saudi Arabia. Data collection was carried out over a 2-month period, from February to April 2025.

### 2.3 Sample size and eligibility

Sample size was determined using *G* ^*^ Power (version 3.1.9.7). Power analyses were conducted for chi-square tests, multiple linear regression, and one-way ANOVA, all assuming an effect size of *f* = 0.20, an alpha level of 0.05, and a statistical power of 0.95. The largest required sample size across these analyses was approximately 246 (one-way ANOVA). A final sample of 420 was selected to enhance generalizability, account for potential missing data, and ensure adequate power for all planned analyses. Inclusion criteria were: Saudi nationals aged 18 years or older, residing in Saudi Arabia, and able to provide informed consent. Non-Saudi residents and individuals with cognitive impairments that could compromise their ability to complete the survey were excluded.

### 2.4 Instrument

The questionnaire comprised three sections. The first section collected sociodemographic data (age, gender, income, area of residence, marital status, educational level, employment status, chronic illness, and medication status). The second section included the HES-A, a validated Arabic-adapted tool assessing perceived health-related empowerment (29). The scale consists of 8 items originally developed for individuals with diabetes (30), rated on a 5-point Likert scale ranging from “strongly disagree” (1) to “strongly agree” (5). Scores were calculated by averaging item responses, with higher means indicating greater perceived health empowerment. The scale demonstrated good validity and reliability (30), with a Cronbach's alpha of 0.92 in the present study. The third section addressed public awareness and engagement with Saudi Arabia's Vision 2030 health initiatives, including healthcare preferences, availability of medical records, participation in health awareness programs, digital health use, and cultural influences.

### 2.5 Statistical analysis

Statistical analyses were conducted using IBM SPSS Statistics (version 27.0). Internal consistency was assessed using Cronbach's alpha, with a minimum threshold of 0.70 considered acceptable. The structural validity of the HES-A was examined via principal components analysis (PCA) with varimax rotation, retaining factors with loadings exceeding 0.40 and eigenvalues >1.00 (31). Chi-square tests were employed to examine associations between categorical variables (32). Multiple linear regression analysis was utilized to investigate the relationships between sociodemographic predictors and the total scores of HES-A. One-way ANOVA was performed to evaluate group differences in HES-A scores for binary categorical variables (gender and residential location) that demonstrated significant bivariate correlations in preliminary analysis. For variables with more than two categories (age group, income, marital status, education, employment status), one-way ANOVA was also conducted but yielded no statistically significant differences (*p* > 0.05 for all); these results are available from the corresponding author upon request. Pearson correlation coefficients were used to assess the relationships between HES-A scores and Vision 2030-related variables. Statistical significance was set at *p* < 0.05.

### 2.6 Ethical considerations

Ethical approval was secured from the Local Research Ethics Committee at the University of Tabuk (Approval No. UT-550-272-2025). The research was conducted in accordance with the principles outlined in the Declaration of Helsinki. All participants provided written informed consent prior to their involvement and were duly informed of their right to withdraw at any point. Confidentiality was rigorously maintained throughout the processes of data collection and analysis.

## 3 Results

### 3.1 Sociodemographic characteristics

The frequency and percentage distributions of sociodemographic factors among the 420 participants are presented in Table 1. The largest age group was 35–44 years (30.7%), followed by 45–54 years (23.6%). Gender distribution was approximately equal (52.4% male; 47.6% female). The majority of participants resided in urban areas (69.8%), were married (65.0%), held a bachelor's degree (56.9%), and were employed (67.6%). Most participants were non-smokers (83.8%), did not report a chronic illness (79.5%), and were not taking regular medication (75.5%).

**Table 1:** Frequencies and percentages for sociodemographic variables (*N* = 420).

Variables	*n*	%
Age group		
18–24	72	17.1
25–34	79	18.8
35–44	129	30.7
45–54	99	23.6
55–64	31	7.4
65–70	10	2.4
Total	420	100.0
Gender		
Female	200	47.6
Male	220	52.4
Total	420	100.0
Monthly income (SAR)		
< 5,000	109	26.0
5,000–10,000	67	16.0
10,001–15,000	110	26.2
>15,000	134	31.9
Total	420	100.0
Area of residence		
Rural	127	30.2
Urban	293	69.8
Total	420	100.0
Marital status		
Single	126	30.0
Married	273	65.0
Divorced	18	4.3
Widowed	3	0.7
Total	420	100.0
Educational level		
Primary school	1	0.2
Intermediate school	4	1.0
High school	31	7.4
Diploma	40	9.5
Bachelor	239	56.9
Master	59	14.0
Doctorate	46	11.0
Total	420	100.0
Employment status		
Student	64	15.2
Employed	284	67.6
Retired	32	7.6
Unemployed	40	9.5
Total	420	100.0
Smoking		
No	352	83.8
Yes	68	16.2
Total	420	100.0
Chronic illness		
No	334	79.5
Yes	86	20.5
Total	420	100.0
On medication		
No	317	75.5
Yes	103	24.5
Total	420	100.0

SAR, Saudi Arabian Riyal.

### 3.2 Correlations between HES-A and sociodemographic variables

Table 2 presents Pearson correlations between HES-A scores and sociodemographic variables. Gender demonstrated a significant positive correlation with health empowerment (*r* = 0.251, *p* < 0.001). A significant negative correlation was observed between residential location and health empowerment (*r* = −0.098, *p* = 0.044), suggesting a potential disparity in perceived empowerment by area of residence. No significant correlations were found between HES-A scores and income, education, chronic illness, or medication use.

**Table 2:** Pearson correlations between the health empowerment scale (HES-A) and sociodemographic variables (*N* = 420).

Variable Pairs	Pearson r	*p*-value
HES—Age	−0.023	0.643
HES—Gender	0.251	< 0.001
HES—Income	−0.052	0.285
HES—Location	−0.098	0.044
HES—Marital Status	0.002	0.970
HES—Education	−0.078	0.110
HES—Employment Status	−0.006	0.902
HES—Smoking	0.087	0.076
HES—Chronic Illness	−0.012	0.808
HES—On Medication	−0.040	0.412

HES-A, Health empowerment scale-Arabic version.

### 3.3 Multiple linear regression: predictors of health empowerment

Multiple linear regression results are presented in Table 3. The overall model was statistically significant [*F*(10, 409) = 3.754, *p* < 0.001] and explained 8.4% of the variance in HES-A scores (*R*^2^ = 0.084; adjusted *R*^2^ = 0.062). Gender was the only significant positive predictor of HES-A scores (β = 0.252, *B* = 0.432, *p* < 0.001), indicating that males reported significantly higher health empowerment than females. Residential location approached statistical significance (*B* = −0.178, *p* = 0.051). No other sociodemographic variable reached significance. According to Cohen's (55) conventions, *f* ^2^ values of 0.02, 0.15, and 0.35 represent small, medium, and large effects, respectively. The overall model accounted for only 8.4% of the variance in HES-A total scores (*R*^2^ = 0.084), yielding a small global effect size (*f* ^2^ = 0.092). This indicates that the sociodemographic variables collectively explain a very limited proportion of health empowerment scores.

**Table 3:** Multiple linear regression: associations of sociodemographic variables with HES-A total scores (*N* = 420).

Predictor	B	SE	β	*t*	*p*	Lower 95% CI	Upper 95% CI	Effect size (Cohen's *f*^2^)
Constant	3.776	0.303	–	12.448	< 0.001	3.180	4.373	–
Age	0.000	0.005	0.005	0.063	0.950	−0.010	0.011	0.000 (negligible)
Gender	0.432	0.081	0.252	5.310	< 0.001	0.272	0.592	0.07100 (small)
Location	−0.178	0.091	−0.093	−1.892	0.051	−0.352	0.007	0.009 (negligible)
Income	−0.037	0.049	−0.051	−0.764	0.446	−0.134	0.059	0.003 (negligible)
Marital status	0.050	0.095	0.033	0.530	0.597	−0.136	0.237	0.001 (negligible)
Education	−0.047	0.045	−0.058	−1.056	0.292	−0.136	0.041	0.003 (negligible)
Employment status	−0.022	0.061	−0.020	−0.368	0.713	−0.141	0.097	0.000 (negligible)
Smoking	0.046	0.112	0.020	0.409	0.683	−0.174	0.266	0.000 (negligible)
Smoking	0.046	0.112	0.020	0.409	0.683	−0.174	0.266	0.000 (negligible)
Chronic illness	−0.071	0.140	−0.034	−0.511	0.610	−0.346	0.203	0.001 (negligible)
On medication	−0.048	0.133	−0.024	−0.359	0.720	−0.309	0.214	0.000 (negligible)

ANOVA: *F*(10, 409) = 3.754, *p* < 0.001. *R*^2^ = 0.084; adjusted *R*^2^ = 0.062. SE, standard error; β, standardized coefficient; CI, confidence interval. Global Effect Size for the Model: Cohen's *f* ^2^ = 0.092 (small effect).

### 3.4 HES-A mean scores across sociodemographic variables

Table 4 presents HES-A mean scores by demographic subgroups. Males reported greater health empowerment than females (mean 4.041 vs. 3.612). Rural participants demonstrated slightly higher empowerment than urban participants (mean 3.965 vs. 3.782). Among age groups, the oldest group (65–70 years) reported the highest mean HES-A score (3.950). Widowed participants reported the highest empowerment by marital status (mean 4.417). Participants without chronic illness or who were not on medication consistently reported higher HES-A scores than their respective counterparts.

**Table 4:** HES-A mean scores across sociodemographic variables (*N* = 420).

Variable/Category	HES mean score
Gender	
Female	3.612
Male	4.041
Area of residence	
Rural	3.965
Urban	3.782
Age group	
18–24	3.825
25–34	3.918
35–44	3.826
45–54	3.768
55–64	3.891
65–70	3.950
Monthly income (SAR)	
< 5,000	3.862
5,000–10,000	3.881
10,001–15,000	3.909
>15,000	3.735
Marital status	
Single	3.799
Married	3.877
Divorced	3.396
Widowed	4.417
Educational level	
Primary school	3.125
Intermediate school	4.000
High school	3.952
Diploma	3.747
Bachelor	3.919
Master	3.623
Doctorate	3.685
Employment status	
Student	3.863
Employed	3.833
Retired	3.805
Unemployed	3.850
Smoking	
No	3.828
Yes	3.881
Chronic Illness	
No	3.865
Yes	3.728
On medication	
No	3.871
Yes	3.733

HES-A, Health Empowerment Scale-Arabic version; SAR, Saudi Arabian Riyal.

### 3.5 Descriptive statistics and psychometric properties of the HES-A

Item-level descriptive statistics for the HES-A are presented in Table 5. Mean scores ranged from 3.74 (self-control and problem-solving items) to 4.00 (support-seeking item; Q6). The composite HES-A score was 3.84 ± 0.855 (skewness = −0.886; kurtosis = 0.238), indicating an approximately normal distribution. Variance inflation factor (VIF) values were all below 4.0, indicating no problematic multicollinearity. Cronbach's alpha for the 8-item scale was 0.92, indicating excellent internal consistency.

**Table 5:** Descriptive statistics of HES-A items (*N* = 420).

Item	Mean ±SD	Skewness	Kurtosis	VIF
Q1. Self-control: awareness of health dissatisfaction	3.74 ± 1.108	−0.836	0.130	2.800
Q2. Self-efficacy: translating health goals into plans	3.77 ± 1.082	−0.825	0.200	3.119
Q3. Problem-solving: overcoming barriers to care	3.74 ± 1.064	−0.803	0.211	3.906
Q4. Psychosocial coping: finding ways to feel better about health	3.85 ± 1.030	−0.922	0.607	3.005
Q5. Stress management: positive coping with health-related stress	3.75 ± 1.036	−0.763	0.131	2.860
Q6. Support: seeking help for health needs	4.00 ± 1.041	−1.062	0.667	1.714
Q7. Motivation: sustaining motivation to care for health	3.93 ± 0.960	−0.964	0.983	3.206
Q8. Decision-making: confidence in personalized health choices	3.92 ± 0.997	−0.946	0.608	3.387
Total HES Score	**3.84** **±0.855**	**−0.886**	**0.238**	**1.021**

HES-A, Health Empowerment Scale-Arabic version; SD, standard deviation; VIF, variance inflation factor.

### 3.6 Score distribution and floor/ceiling effects

Score distributions for HES-A items are presented in Table 6. Most participants rated their health empowerment positively, with best-result scores (4, 5) ranging from 66.2% to 75.7% across items. Floor effects were minimal across all items ( ≤ 5.7%). Item 6 (support-seeking) exhibited the highest ceiling effect (37.1%), suggesting that the ability to seek support for health needs was widely endorsed.

**Table 6:** Score distributions for HES-A items: floor and ceiling effects (*N* = 420).

Score category	Item 1	Item 2	Item 3	Item 4	Item 5	Item 6	Item 7	Item 8	Total HES
Worst result (% scoring 1–3)	33.3	33.3	33.8	30.0	32.9	24.3	26.7	26.2	30.06
Best result (% scoring 4–5)	66.7	66.7	66.2	70.0	67.1	75.7	73.3	73.8	69.94
Floor effect (% scoring 1)	5.7	5.0	4.8	4.3	3.6	3.3	3.1	2.9	4.09
Ceiling effect (% scoring 5)	26.7	27.4	25.0	28.3	24.5	37.1	29.5	30.7	28.65

HES-A, Health Empowerment Scale-Arabic version. Worst result = % scoring 1–3; Best result = % scoring 4–5; Floor effect = % scoring 1; Ceiling effect = % scoring 5.

### 3.7 Gender differences in self-perceived health management and decision-making

Table 7 presents chi-square analyses of HES-A item responses by gender. Significant gender differences were observed across all eight items (all *p* ≤ 0.035). Males consistently reported higher levels of strong agreement across items measuring health awareness [Q1: χ^2^(4) = 10.333, *p* = 0.035], goal-setting [Q2: χ^2^(4) = 10.876, *p* = 0.028], barrier management [Q3: χ^2^(4) = 11.371, *p* = 0.023], psychosocial coping [Q4: χ^2^(4) = 17.786, *p* = 0.001], stress coping [Q5: χ^2^(4) = 20.120, *p* < 0.001], support-seeking [Q6: χ^2^(4) = 13.268, *p* = 0.010], motivation [Q7: χ^2^(4) = 22.442, *p* < 0.001], and decision-making [Q8: χ^2^(4) = 15.340, *p* = 0.004]. One-way ANOVA confirmed a significant overall gender difference in HES-A scores [*F*(1, 418) = 28.112, *p* < 0.001].

**Table 7:** Gender differences in HES-A item responses (chi-square tests; *N* = 420).

Item/Gender	1	2	3	4	5	Total	χ^2^	p
Q1. Awareness of health dissatisfaction								
Female	9	15	40	94	42	200	10.333	0.035
Male	15	18	43	74	70	220		
**Total**	24	33	83	168	112	420		
Q2. Health goals into workable plan								
Female	10	12	44	92	42	200	10.876	0.028
Male	11	18	45	73	73	220		
**Total**	21	30	89	165	115	420		
Q3. Overcoming barriers to care goals								
Female	7	16	46	94	37	200	11.371	0.023
Male	13	16	44	79	68	220		
**Total**	20	32	90	173	105	420		
Q4. Finding ways to feel better about health								
Female	6	8	40	103	43	200	17.786	0.001
Male	12	14	46	72	76	220		
**Total**	18	22	86	175	119	420		
Q5. Positive coping with health-related stress								
Female	9	19	42	100	30	200	20.120	< 0.001
Male	6	19	43	79	73	220		
**Total**	15	38	85	179	103	420		
Q6. Seeking support for health needs								
Female	3	14	29	92	62	200	13.268	0.010
Male	11	14	31	70	94	220		
**Total**	14	28	60	162	156	420		
Q7. Motivation to maintain health								
Female	4	7	46	104	39	200	22.442	< 0.001
Male	9	9	37	80	85	220		
**Total**	13	16	83	184	124	420		
Q8. Healthcare decision-making								
Female	4	13	41	97	45	200	15.340	0.004
Male	8	15	29	84	84	220		
**Total**	12	28	70	181	129	420		
ANOVA: DV = HES; IV = Gender								
**Source**	**SS**	**df**	**MS**	***F***	***p***			
Between Groups	19.335	1	19.335	28.112	< 0.001			
Within Groups	287.493	418	0.688					
Total	306.828	419						

Likert scale: 1 = strongly disagree; 5 = strongly agree. χ^2^, chi-square statistic; df, degrees of freedom; SS, sum of squares; MS, mean square.

### 3.8 Impact of residence on health knowledge, motivation, and coping

Table 8 presents chi-square analyses of HES-A item responses by area of residence. Significant differences between rural and urban residents were observed for awareness of healthcare dissatisfaction [Q1: χ^2^(4) = 11.074, *p* = 0.026] and motivation to maintain health [Q7: χ^2^(4) = 16.309, *p* = 0.003]. No significant differences were found for the remaining items, suggesting that coping mechanisms, goal-setting capacity, support-seeking behavior, and healthcare decision-making are broadly comparable across residential settings. The overall HES-A score was marginally higher among rural residents [mean 3.965 vs. 3.782; *F*(1, 418) = 4.081, *p* = 0.044].

**Table 8:** Impact of residential location on HES-A item responses (chi-square tests; *N* = 420).

Item/Residence	1	2	3	4	5	Total	χ^2^	*p*
Q1. Awareness of health dissatisfaction								
Rural	5	10	17	49	46	127	11.074	0.026
Urban	19	23	66	119	66	293		
**Total**	24	33	83	168	112	420		
Q2. Health goals into workable plan								
Rural	2	11	21	53	40	127	8.124	0.087
Urban	19	19	68	112	75	293		
**Total**	21	30	89	165	115	420		
Q3. Overcoming barriers to care goals								
Rural	5	6	28	48	40	127	5.877	0.209
Urban	15	26	62	125	65	293		
**Total**	20	32	90	173	105	420		
Q4. Finding ways to feel better about health								
Rural	5	8	20	50	44	127	5.221	0.265
Urban	13	14	66	125	75	293		
**Total**	18	22	86	175	119	420		
Q5. Positive coping with health-related stress								
Rural	3	11	22	55	36	127	2.645	0.619
Urban	12	27	63	124	67	293		
**Total**	15	38	85	179	103	420		
Q6. Seeking support for health needs								
Rural	6	8	12	51	50	127	4.437	0.350
Urban	8	20	48	111	106	293		
**Total**	14	28	60	162	156	420		
Q7. Motivation to maintain health								
Rural	2	7	25	41	52	127	16.309	0.003
Urban	11	9	58	143	72	293		
**Total**	13	16	83	184	124	420		
Q8. Healthcare decision-making								
Rural	3	8	17	50	49	127	5.624	0.229
Urban	9	20	53	131	80	293		
**Total**	12	28	70	181	129	420		
ANOVA: DV = HES; IV = Location								
Source	SS	df	MS	F	p			
Between Groups	2.967	1	2.967	4.081	0.044			
Within Groups	303.861	418	0.727					
Total	306.828	419						

Likert scale: 1 = strongly disagree; 5 = strongly agree. χ^2^, chi-square statistic; SS, sum of squares; MS, mean square.

### 3.9 Vision 2030 health engagement and healthcare preferences

Table 9 presents participants' engagement with Vision 2030 health initiatives and associated correlations with HES-A scores. The majority of participants (56.4%) preferred government hospitals and health centers for medical issues. A significant negative correlation was observed between HES-A scores and preference for government-based medical consultation (*r* = −0.122, *p* = 0.012), suggesting that individuals with higher health empowerment may favor self-care or alternative health management strategies. The majority of participants (71.7%) maintained an active medical record at a local health facility. Participation in community health programs was reported by 44.5% of participants and was negatively correlated with HES-A scores (*r* = −0.106, *p* = 0.030), potentially indicating that highly empowered individuals access alternative health resources. Three-quarters of participants (75.0%) used digital health platforms (Seha, Mawid), although 24.9% used them rarely or never. Awareness of government initiatives supporting women's health was reported by 66.2% of participants. Cultural norms were perceived as positively influencing healthcare-seeking behavior by 58.3% of participants, while 10.2% reported a negative cultural influence and 31.4% reported no effect.

**Table 9:** Public engagement with Vision 2030 health initiatives and Pearson correlations with HES-A scores (*N* = 420).

Item	*n*	%	Pearson *r*	*p-*value
Healthcare preference (preference for medical consultation)	−0.122	0.012		
Government hospitals/health centers	237	56.4		
Does not visit hospitals/health centers	41	9.8		
Private hospitals/health centers	142	33.8		
Total	420	100.0		
Active medical record at local health center				
No	119	28.3	0.048	0.322
Yes	301	71.7		
Total	420	100.0		
Participation in community health programmes (e.g., screenings, campaigns)				
No	233	55.5	−0.106	0.030
Yes	187	44.5		
Total	420	100.0		
Frequency of digital health platform use (Seha, Mawid)				
Always	156	37.1	−0.047	0.333
Sometimes	159	37.9		
Rarely	95	22.6		
Never	10	2.4		
Total	420	100.0		
Awareness of government initiatives for women's health				
No	142	33.8	0.047	0.338
Yes	278	66.2		
Total	420	100.0		
Cultural norms and healthcare-seeking behavior				
No effect	132	31.4	−0.006	0.897
Yes, negatively	43	10.2		
Yes, positively	245	58.3		
Total	420	100.0		

HES-A, Health Empowerment Scale-Arabic version. Correlation coefficients refer to the association between each item category and total HES-A scores.

## 4 Discussion

This study examined health empowerment across sociodemographic groups in Saudi Arabia using the HES-A and explored associations with Vision 2030 health engagement indicators. The findings contribute to an emerging evidence base on the determinants of health empowerment in the Arabian Gulf context.

### 4.1 Gender and health empowerment

A significant positive correlation between gender and health empowerment (*r* = 0.251, *p* < 0.001) was observed, with males exhibiting higher HES-A scores than females. This result aligns with the findings of King et al. (33), who emphasize the beneficial impact of gender equity on health outcomes across all genders, particularly within workplace settings. Nonetheless, the current investigation also revealed that females demonstrated greater familiarity with coping strategies and expressed higher comfort levels in seeking assistance, which may reflect ongoing societal reforms within Saudi Arabia's healthcare sector (34, 35). Gender disparities in self-perceived health autonomy are extensively documented and are especially prominent in contexts where healthcare systems or sociocultural norms have historically limited women's agency (5). The present results accentuate the necessity of implementing targeted interventions aimed at improving women's health literacy, facilitating access to healthcare services, and boosting confidence in navigating healthcare systems.

### 4.2 Residential location and health empowerment

Residential location substantially influenced health knowledge, motivation, and coping strategies. Rural participants reported marginally higher overall HES-A scores (mean 3.965 vs. 3.782, *p* = 0.044) and exhibited significantly greater motivation for health maintenance (Q7: *p* = 0.003). These findings may indicate enhanced intrinsic motivation or reliance on self-directed care among rural residents, a pattern supported by Alfaqeeh et al. (15), who observed that rural respondents in Riyadh Province exhibited higher education and income levels than anticipated. Elsheikh et al. (36) further reported improved healthcare access in remote regions attributable to facility expansion, and the widespread adoption of telemedicine, electronic health records, and artificial intelligence within Saudi healthcare (37) may further mitigate traditional rural–urban disparities. Nonetheless, the near-significant association between residential location and regression-adjusted HES-A scores (*p* = 0.051) warrants further exploration, as urban residents may encounter fragmented care or reduced self-efficacy despite greater service availability.

The observation that residents in rural areas report higher levels of health empowerment compared to their urban counterparts appears to contradict the statement in the introduction indicating that rural populations have 40% lower rates of specialist consultation. However, these findings assess different constructs and are not mutually exclusive. Health empowerment pertains to an individual's perceived control, self- efficacy, and motivation to manage their health—psychological assets that may be enhanced by necessity in resource- constrained environments. Conversely, health service utilization reflects actual access to and use of formal healthcare services, which can be limited by factors such as geographic distance, shortages of providers, and transportation costs, irrespective of psychological empowerment. This pattern of increased access barriers among rural populations is documented within the Saudi context itself by Alfaqeeh et al. (15), who reported that respondents from rural areas in Riyadh Province were significantly more likely than their urban counterparts to cite distance to the primary health care center as an obstacle (28.0% vs. 12.9%, *p* < 0.001). Furthermore, they were markedly less likely to have undergone a medical investigation in the preceding year. At the national level, Al-Hanawi and Chirwa (38), utilizing data from the 2018 Saudi Family Health Survey (*N* = 11,528), similarly observed that the utilization of preventive health check-ups was predominantly concentrated among wealthier and more educated Saudi citizens, with the extent of socioeconomic inequality varying substantially across the administrative regions of the Kingdom. It is important to emphasize that neither Alfaqeeh et al. (15) nor Al-Hanawi and Chirwa (38) directly evaluated health empowerment or motivation; their findings primarily underscore the structural barriers to access and the socioeconomic disparities in engagement with formal preventive care observed in the Saudi population. Conversely, the higher levels of health empowerment and motivation among rural participants are specific to the results of the current study.

Notably, Alfaqeeh et al. (15) also found that, despite these access barriers, rural respondents were significantly more likely than urban respondents to want advice on healthy eating, even though they were less likely to actually receive it, suggesting that motivation to engage with preventive health information can coexist with, rather than be eliminated by, reduced access to formal care. In the current study, rural respondents scored significantly higher on motivation for health maintenance (Q7: 40.9% rural vs. 24.6% urban scored 5) and reported marginally higher overall HES-A scores, indicating that the presence of more access barriers does not preclude heightened perceived self-efficacy or motivation—constructs that are distinct from actual service utilization. Although this pattern is broadly comparable to that reported by Alfaqeeh et al. (15) within the same Saudi rural-urban context, the magnitude of the rural–urban difference observed in the current sample is modest, and the present data do not permit a detailed test of the mechanisms underlying it. One possible explanation, which warrants further investigation, is that dissatisfaction with the limited healthcare infrastructure available in rural areas of Saudi Arabia may itself heighten awareness of health needs and motivation to address them; this possibility should be examined directly, alongside relevant data on the current rural healthcare landscape, in future research.

### 4.3 Healthcare preferences and institutional engagement

The majority of participants (56.4%) preferred government health facilities, while 33.8% favored private facilities. A negative correlation between preference for government-based consultations and HES-A scores (*r* = −0.122, *p* = 0.012) may indicate dissatisfaction with the quality of public-sector services. Almalki et al. (39) report that users of Saudi government health facilities frequently cite long wait times and inconsistent quality of care, which may reduce perceived autonomy and control over healthcare decisions (15, 40). Patient empowerment is inherently linked to access to and continuity of care; notably, 71.7% of participants possessing an active medical record demonstrate a high level of engagement with formal healthcare services, aligning with the objectives of integrated e-health systems as outlined in Vision 2030 (41). International evidence indicates that the preference for public vs. private healthcare is associated with waiting times in publicly funded systems, with the strength of this association differing by country depending on factors such as public-sector capacity, private insurance coverage, and patient dissatisfaction. In certain OECD health systems, extended wait times for non-urgent specialist and elective treatments compel wealthier patients to seek private care rather than endure delays on public waiting lists (42). However, this is not universal, as OECD countries exhibit variability in median wait times for procedures such as hip and knee replacements, ranging from approximately 50 days or fewer in Denmark, Hungary, Italy, and the Netherlands to more than 240 days in Estonia and Chile (43). A study conducted among emergency department patients in the Northern Emirates of the United Arab Emirates revealed that individuals receiving treatment at private hospitals reported significantly higher satisfaction levels in comparison to those treated at public hospitals. Waiting time was identified as the principal cause of dissatisfaction with public-sector healthcare services (44). These international patterns align with the current finding that dissatisfaction correlates with lower HES-A scores among individuals who prefer government-based consultations.

### 4.4 Community program participation and digital health

Only 44.5% of participants had engaged in community health programs, and non-participation was significantly associated with higher HES-A scores (*r* = −0.106, *p* = 0.030). This suggests that highly empowered individuals may access health information and resources independently, potentially bypassing structured community programs (45). Digital health platforms were used by 75.0% of participants, consistent with Saudi Arabia's Ministry of Health expansion of e-health services, including Sehhaty, Mawid, Tabaud, Sehha, and Wasfaty (46, 47).

Nevertheless, 24.9% of individuals reported infrequent or no utilization of digital health tools. This absence of use may be attributable to various factors: lack of awareness, perceived irrelevance, preference for face-to-face consultations, or genuine barriers such as digital literacy and access issues. Further research is necessary to differentiate among these potential explanations. Addressing these gaps is a key objective of Vision 200′s digital transformation agenda, though concerns remain about the adequacy and reliability of available health information (46, 48) and the estimated 46% prevalence of low health literacy among Saudis (49).

### 4.5 Cultural influences on healthcare-seeking behavior

The majority of participants (58.3%) perceived cultural norms as positively influencing their healthcare-seeking behavior, while 10.2% reported a negative effect and 31.4% reported no effect. Cultural norms play a complex role in shaping health empowerment in Saudi Arabia; while traditional values may support community health engagement, some telehealth interventions have been found to be culturally inappropriate, limiting widespread adoption (50). Some telehealth interventions are culturally inappropriate in the Saudi context because they may require women to appear without hijab during video consultations, limit family participation in healthcare decisions, or use non-Arabic and impersonal communication systems that conflict with traditional relationship-based care. These cultural mismatches can discourage telehealth use despite adequate access and digital literacy (51–53). This is particularly relevant in the context of Vision 200′s emphasis on patient-centered digital healthcare. Communication barriers, particularly for non-Arabic-speaking health workers, may further affect the quality of patient–provider interactions and perceived empowerment (54).

## 5 Limitations

This study has several limitations that should be considered when interpreting the findings. The cross-sectional design prevents causal inference; for instance, it remains unclear whether lower empowerment leads to the avoidance of community programs or vice versa. Although the sample size was statistically sufficient, recruitment through social media platforms may introduce self-selection bias and restrict the generalizability of the results to populations with limited digital access, including older adults and individuals living in remote regions. The study did not directly assess technological literacy or internet accessibility, both of which may impact engagement with e-health tools and perceptions of health empowerment. Future longitudinal and mixed-methods research should address these limitations and investigate additional determinants, such as stress, physical activity, and mental health status. Our findings may be relevant to digitally connected adults of working age; however, they may not fully capture the experiences of older adults, residents of remote areas without reliable internet access, or individuals with limited digital literacy and access to social media.

## 6 Conclusion

This study demonstrates that gender and residential location are the primary sociodemographic factors influencing self-perceived health empowerment in Saudi Arabia. Males reported significantly higher HES-A scores than females across all dimensions of the scale, while rural residents demonstrated marginally higher overall empowerment, with notably greater motivation for health maintenance. Income, educational attainment, chronic illness, and other demographic variables were not significantly associated with health empowerment in this sample. Engagement with Vision 2030 health initiatives was generally high, with strong preference for government health facilities, widespread use of digital health platforms, and broadly positive perceptions of cultural influences on healthcare-seeking behavior. The findings underscore the need for targeted, gender-responsive, and culturally sensitive public health interventions to address disparities in health empowerment and to advance equitable health service utilization in Saudi Arabia. Policymakers and healthcare providers should implement targeted, gender-responsive health programs, expand community-health-worker and digital health platforms in rural areas, strengthen continuity of care in urban centers, and include digital-literacy training in digital-health initiatives to bridge gender and urban-rural gaps in health empowerment.

## 7 Implications for policy and practice

The significant gender-based differences in health empowerment dimensions identified in this study underscore the need for targeted interventions to enhance women's health literacy, access to services, and confidence in navigating healthcare systems. In Saudi Arabia, where gender roles are shifting under Vision 2030, this finding highlights the importance of gender-responsive health service design and community outreach programs tailored to women's specific health needs and barriers to empowerment. The differences between rural and urban residents in health dissatisfaction awareness and motivation suggest a need for context-specific strategies. Urban interventions might prioritize informational and relational support to address fragmented care, whereas rural approaches should reinforce self-care competencies through community health workers and digital platforms. In alignment with Vision 2030, the observed associations between institutional healthcare preferences, community program non-participation, and lower HES-A scores support increased investment in public sector service delivery, comprehensive awareness campaigns, and community outreach tailored to underserved and marginalized populations.

## Data Availability

The raw data supporting the conclusions of this article will be made available by the authors, without undue reservation.
